# Utility of PD-L1 immunohistochemistry assays for predicting PD-1/PD-L1 inhibitor response

**DOI:** 10.1186/s40364-017-0093-8

**Published:** 2017-03-15

**Authors:** Laurence P. Diggs, Eddy C. Hsueh

**Affiliations:** Division of General Surgery, Department of Surgery, Saint Louis University, 3635 Vista at Grand Blvd., St. Louis, MO 63110 USA

**Keywords:** Metastatic melanoma, Non-Small-Cell Lung Cancer, PD-1/PD-L1 inhibitors, PD-L1 immunohistochemistry assays

## Abstract

We have seen a notable increase in the application of PD-1/PD-L1 inhibitors for the treatment of several solid and hematogenous malignancies including metastatic melanoma, non-small-cell lung cancer and lymphoma to name a few. The need for biomarkers for identification of a suitable patient population for this type of therapy is now pressing. While specific biomarker assays have been developed for these checkpoint inhibitors based on their respective epitopes, the available studies suggested the clinical utility of these biomarker assays is for response stratification and not patient selection. Further improvement in assay development is needed to utilize this type of assay in identification of ideal patient population for this therapy.

## Introduction

Immune modulation therapies have seen an impressive growth over the last decade [1]. Recently, inhibitors of programmed cell-death receptor (PD-1) and its associated ligand (PD-L1) have gained significant attention from the oncology community. PD-L1, typically expressed on the surface of healthy cells, binds PD-1 on primed cytotoxic T cells thereby inhibiting cell-mediated attack [1–3]. Multiple studies reported worse outcomes in tumors expressing PD-L1 [2, 3]. Purportedly, the expression of this ligand on tumor cells confers protection against immune-mediated attacks on tumor cells and may account for their particularly malignant potential. Anti-PD-L1 (or anti-PD-1) monoclonal antibodies inhibit PD-L1 binding to PD-1 and allow T cell activity at this immune checkpoint. Several clinical trials using these antibodies for the treatment of malignancies such as melanoma, non-small-cell lung cancer (NSCLC), head and neck cancer, renal cell cancer, urothelial cancer and lymphoma have shown great promise in prolonging survival [4–10].

However, not all patients respond to PD-1/PD-L1 inhibitors. Thus, predicting the likelihood of response to treatment would aid in appropriate patient selection for these drugs. Immunohistochemistry (IHC) biomarker assays for respective PD-1/PD-L1 inhibitors were designed to screen for the presence of specific PD-1/PD-L1 epitopes as well as to estimate the percentage of T cells or tumor cells expressing this receptor or ligand. At this time, 4 FDA-approved IHC biomarker assays have been designed [10]. Their ability to consistently and reproducibly quantify proportion of cells expressing PD-L1 has been evaluated in prospective trials. Given the inherent heterogeneity of gene expression between individual tumors and among tumor cells within the same tumor nodules, there are concerns that any single assay using a fixed percentage of PD-L1 positive tumor cells could accurately determine the appropriate patients for treatment [11, 12]. This is reflected in the finding that PD-1/PD-L1 inhibitors appear to have activity in a subset of individuals who do not meet the IHC bioassay cutoff. Furthermore, recent studies suggested that several additional factors could be involved in the response to anti-PD-1/PD-L1 antibodies.

## Current PD-1 & PD-L1 inhibitors

Among the first generation of these drugs, Pembrolizumab (an anti PD-1 antibody) was approved for treatment of NSCLC [13–16] and melanoma [17–20] in 2014. Pembrolizumab has also recently been approved for use in advanced head and neck squamous cell carcinoma (HNSCC) [21, 22]. Nivolumab (an anti PD-1 antibody) was approved for melanoma in 2014 [23–28] NSCLC in 2015 [29–32] and renal cell carcinoma in 2015 [33, 34]. Pembrolizumab and Nivolumab have been demonstrated to improve overall and progression free survival in the above-mentioned tumors. Atezolizumab (an anti PD-L1 antibody) has received FDA designated breakthrough drug status for two malignancies. Clinical trials are currently underway for both metastatic NSCLC [35–37] and for urothelial carcinoma [38]. Durvalumab (an anti-PD-L1 antibody) is also being evaluated in clinical trials for the treatment of NSCLC (phase III) [39] and bladder cancer (phase III) [40, 41]. Pidilizumab (an anti PD-1 antibody) is currently being tested in the treatment of large B cell lymphoma (Phase II completed) [42]. Finally, Avelumab (an anti PD-L1 antibody) is currently being tested in patients with Merkel cell carcinoma (Phase II) [43] and NSCLC (Phase III) [44].

## Current PD-1/PD-L1 bioassays

Several studies examining the usefulness of PD-L1 IHC assays have demonstrated a direct correlation of response rate to PD-L1 expression level. The distinction between a companion assay and a complementary assay should be underlined here. A companion assay is one that is considered to be essential to the use of its corresponding drug. Pembrolizumab is FDA approved only when used in conjunction with the Dako 22C3. Conversely, the other bioassays are considered complementary in that their use is recommended in order to optimize appropriate patient selection but is not considered mandatory for the use of its associated drug [45]. The cutoff values for these assays vary from as low as 1% to as high as 50%. To allow for comparison, sensitivity (SENS) and specificity (SPEC) of the bioassay for a given malignancy were calculated based on the reported objective response rate in individuals who were considered to have PD-L1 positive tumors (ORR+) and that of the individuals who were considered to have PD-L1 negative tumors (ORR-). Sensitivity was calculated as a ratio of true positives (ORR+) to the sum of true positives (ORR+) and False Negatives (ORR-). Specificity was calculated as a ratio of true negatives (1-ORR-) to the sum of true negatives (1-ORR-) and false positives (1-ORR+).

Pembrolizumab is currently approved for the treatment of NSCLC, advanced HNSCC and advanced melanoma. Its companion IHC biomarker assay, Dako 22C3, is used to detect PD-L1 in all three types of malignancies. It is the only assay that has FDA companion status [45]. This exceptional status is due to the assays reliability when testing for PD-L1 positivity making it an essential tool when assessing which candidates are appropriate for treatment with Pembrolizumab. The Dako 22C3 PD-L1 positivity cutoff is 1% for melanoma. The average ORR+ is 39% for PD-L1 positive tumors and the average ORR- is 10% for PD-L1 negative tumors. These estimates are based on the findings from Daud et al. [19] who graded PD-L1 positivity and negativity based on the MEL score. Using a 1% expression as a cutoff, MEL scores of 0 and 1 were considered negative whereas MEL scores of 2,3,4 and 5 were considered positive. The ORR- and ORR+ were weighted averages of MEL 0 and 1 and MEL 2–5 respectively. The associated SENS and SPEC for this bioassay are 80 and 60%, respectively. The cutoff for HNSCC PD-L1 positivity is also 1%. The ORR+ and ORR- are 22% and 4%, respectively [21, 22]. SENS is 85% and SPEC is 55%. For NSCLC, the Dako 22C3 PD-L1 positivity cutoff is 50%. ORR+ and ORR- are 41 and 13%, respectively [13–15]. The SENS is 76% and the SPEC is 60% (Table 1).

**Table 1:** Biomarker assays for anti-PD-1/PD-L1 drugs and associated outcomes

Bioassay	Drug	Disease Target	Cut Off^b^	ORR+^c^	ORR-^d^	SENS^e^	SPEC^f^
Roche Ventana SP263	Durvalumab	NSCLC^a^	25%	27%	5%	84%	78%
Roche Ventana SP263	Durvalumab + Tremelimumab	NSCLC	25%	22.5%	29%	36%	48%
Roche Ventana SP263	Durvalumab	Bladder Cancer	25%	46%	0%	100%	65%
Roche Ventana SP142	Atezolizumab	Metastatic NSCLC	50%	45%	14%	76%	61%
Roche Ventana SP142	Atezolizumab	Urothelial Carcinoma	1%	27%	13%	NA	NA
Dako 22C3	Pembrolizumab	NSCLC	50%	41%	13%	76%	60%
Dako 22C3	Pembrolizumab	HNSCC	1%	22%	4%	85%	55%
Dako 22C3^g^	Pembrolizumab	Melanoma	1%	39%	10%	80%	60%
Dako 28-8	Nivolumab	Non-Squamous NSCLC	1%	19%	9%	68%	53%
Dako 28-8	Nivolumab	Squamous NSCLC	5%	20%	20%	NA	NA
Dako 28-8	Nivolumab	Melanoma	5%	57%	41%	58%	49%
Dako 28-8	Nivolumab + Ibilimumab	Melanoma	5%	72%	55%	57%	54%

^a^NSCLC: Non-Small-Cell Lung Cancer – ^b^Cut Off: Proportion of tumor cells expressing PD-L1 below which tumor is considered PD-L1 negative. – ^c^ORR+ Objective Response Rate in PD-L1 positive tumors. – ^d^ORR-: Objective Response Rate in PD-L1 negative tumors. – ^e^Sensitivity of bioassay for predicting response to PD-L1 blockade based on established cut off. ^f^Specificity of bioassay for predicting response to PD-L1 blockade based on established cut off. – ^g^ORR+ and ORR- based on weighted average of corresponding MEL scores

Nivolumab is currently approved for the treatment of squamous and non-squamous NSCLC, advanced RCC and advanced melanoma. Its companion PD-L1 IHC biomarker assay, Dako 28–8, is only used in tumor tissue from NSCLC and melanoma [46]. In the case of RCC, the PD-L1 expression detected on Dako 28–8 was not predictive of response to Nivolumab [33, 34]. Nivolumab is considered second line therapy for RCC regardless of PD-L1 status. For melanoma, the Dako 28–8 PD-L1 positivity cutoff is 5%. The ORR+ was 57% and the ORR- was 41% and the associated SENS and SPEC are 58 and 49%. An interesting set of findings was brought about when Nivolumab was combined with Ipilimumab, an anti- CTLA4 antibody. The ORR+ and ORR- are 72 and 55%, respectively and the associated SENS and SPEC for this bioassay are 57 and 54%, respectively [25, 27, 28]. The Dako 28–8 cutoff for NSCLC is 1%. For non-squamous NSCLC, the ORR+ and ORR- are 19 and 9%, respectively [32]. SENS is 68% and SPEC is 53%. For squamous NSCLC, there was no significant difference between ORR+ and ORR- which could be estimated at approximately 20% [30, 31]. The SENS and SPEC could therefore not be calculated (Table 1).

Durvalumab is currently approved for the treatment of NSCLC and bladder cancer. Its companion IHC biomarker assay is Roche Ventana SP263 [47]. The SP263 PD-L1 positivity cutoff is 25% for NSCLC. The ORR is 27% for PD-L1 positive tumors and 5% for PD-L1 negative tumors [39]. The associated SENS and SPEC are 84 and 78%, respectively. A recent study compared Durvalumab alone to combination therapy with Durvalumab and Tremelimumab (an anti CTLA-4 antibody). The ORR+ and ORR- were 22.5 and 29% respectively indicated that the ORR appeared to be negatively affected by higher PD-L1 expression. The SENS and SPEC were 36 and 48%. The SP263 cutoff for bladder cancer is also 25%. The ORR+ and ORR- are 46 and 0%, respectively [40, 41] with an associated SENS of 100% and SPEC of 65% (Table 1).

Atezolizumab is currently approved for treatment of metastatic NSCLC and urothelial cancer [35]. Its companion IHC biomarker assay is Roche Ventana SP142. The SP142 PD-L1 positivity cutoff is 50% for NSCLC. ORR+ is 45% and ORR- is 14% [36, 37]^.^ The associated SENS and SPEC are 76 and 61%, respectively. The cutoff for urothelial cancer is 1%. The ORR+ and ORR- are 27% and 15%, respectively [38]. However, in this study, the ORR- included both patients with IHC staining <1% and patients with IHC staining between 1 and 5%. Therefore, the SENS and SPEC for SP142 in urothelial cancer could not be calculated (Table 1).

In general, the average specificity for these assays is 58%. Thus, approximately 42% of patients who are not likely to respond to treatment are considered PD-L1+. Furthermore, the average overall sensitivity of these assays with their respective cutoff levels is 72%; suggesting an average of 28% of patients who are considered PD-L1 negative may benefit from this type of treatment. The estimates are based on the figures seen in table.

## Factors influencing PD-L1 expression

The mechanism of PD-L1 expression is complex. Several factors appear to influence both PD-L1 expression and response to treatment [48–52]. BRAF and MEK mutations contribute to dysfunction of the Ras-Raf-MEK-ERK Map kinase mutations that are present in greater than 90% of melanomas. Specific BRAF mutations when pretreated with Dabrafenib have been associated with reduced response to PD-1/PD-L1 inhibition in melanoma [53–55]. However, when Dabrafenib was combined with MEK suppressor Tremelimumab, an improved response to PD-L1 inhibition was noted [55, 56]. Similarly, blockade of mutated BRAF and MEK was associated with improved response to PD-1/PD-L1 inhibition in NSCLC. Another interesting relationship is the one between mutations in EGFR and EML4-ALK and the expression of PD-L1. Recent studies indicate that EGFR mutations and rearrangements in EML4-ALK are associated with up regulation of PD-L1 synthesis and expression in NSCLC [57–59]. This was further established when patients with these mutations were treated with tyrosine kinase inhibitors and exhibited in lower overall response to PD-L1 blockade [60]. The presence of KRAS in the tumor also appears to be associated with increased expression of PD-L1 [60]. In a case report, KRAS pretreatment is reported to have increased response to Nivolumab in a patient who had not responded to several other treatment courses [61]. In addition, PD-L1 expression can increase due to local pro-inflammatory factors. Cigarette smoking in patients with NSCLC appears to increase the number of lymphocytes present as well as the overall proportion of PD-L1 present [62]. Platinum based chemotherapy [63] also appears to affect the tumor environment in a similar way to cigarette smoke. Additional studies have attributed tumor resistance to immunotherapies to immunosuppressive events occurring in the tumor microenvironment. Several of the mechanisms have been studied in clinical samples and validated in mouse models. The most important may be extrinsic suppression of CD8+ effector cells by CD4 + CD25 + FoxP3+ regulatory T cells (Tregs) [64, 65]. The metabolic deregulation via tryptophan catabolism by indoleamine-2,3-dioxygenase (IDO) may also play a role [66]. With multiple intrinsic and extrinsic factors influencing PD-L1 expression, PD-L1 expression may vary over time. Since tumor cells can exert an adaptive immune response over time, tumor tissue may express little PD-L1 at the moment of tissue sampling for IHC staining but expression may increase considerably at later time point in the disease course [52, 67, 68].

## Conclusion

Modification of specific checkpoints in anti-tumor immune response has resulted in significant improvement for the treatment of various malignancies. The relationship between tumor expression of PD-L1 and patient outcome has been established. However, the currently available IHC biomarker assays could not provide clinically meaningful identification of responders and non-responders [11, 12, 67, 68]. The sensitivity and specificity of the IHC assays are generally poor. The stringent application of the results of these assays would exclude up to 28% of individuals who may benefit from treatment and include up to 42% of patients who may not benefit.

Several tumor and patient characteristics appear to influence response to PD-1/PD-L1 inhibitor and should be considered when selecting patients for this treatment. Providing several tissue samples and obtaining tissue samples at different time intervals may allow for more accurate determination of appropriate patient for treatment.

A direct comparison of the clinical utility of these diagnostic assays for lung cancer was recently completed. In the Blueprint PD-L1 IHC Assay Comparison Project, the Pathology Committee of the International Association for the Study of Lung Cancer joined efforts with 6 of the commercial stakeholders (Astra Zeneca, Bristol Myers Squibb, Dako, Merck Sharpe Dohme, Roche/Genentech Pharmaceuticals, and Roche Ventana Diagnostics) to compare these tests. 3 of the 4 assays showed similar results but the SP142 demonstrated significantly less expression [72]. The interchangeability of the current assays is likely to be a challenge [69–71]. One promising strategy is the study of mRNA via in situ hybridization aimed at providing useful data for predicting success of these drugs on their targets [73].
